# Performance evaluation of an automatic chemiluminescence immune platform for SARS-CoV-2 neutralizing antibody after vaccination in real world

**DOI:** 10.1186/s12879-022-07141-8

**Published:** 2022-02-15

**Authors:** Min Li, Ruiwei Jiang, Enyun Wang, Dan Xiong, Tong Ou, Xiuming Zhang, Xiaowen Dou

**Affiliations:** 1 Medical Laboratory of Shenzhen Luohu People’s Hospital, Shenzhen, 518001 China; 2 Medical College, Anhui University of Science and Technology, Huainan, 232000 Anhui China

**Keywords:** SARS-CoV-2 vaccine, Immunoassays, Neutralizing antibodies, Serological assay, Plaque reduction neutralization test, Real world

## Abstract

**Objective:**

Reliable high-throughput serological assays for SARS-CoV-2 antibodies present an important role in the strength and duration of immunity after vaccination. The study investigated the analytical and clinical performances of neutralizing antibodies (NTAb) assay by chemiluminescent (CLIA), and SARS-CoV-2 neutralizing antibody after vaccination in real world.

**Methods:**

The analytical performances of CLIA for SARS-CoV-2 NTAb were evaluated, followed by the sensitivity and specificity identified with a PRNT test from 50 volunteers. Then, a cohort of vaccine recipients (*n* = 37) were tracked with SARS-CoV-2 NTAb assay at prior to vaccination, one, three and six months post two doses. In real world, a total of 737 cases were recruited from physical examination center in Shenzhen Luohu People’s Hospital (from Jun to August 2021) to analyze vaccination status.

**Results:**

Serological assays on the CLIA were found with excellent characteristics including imprecision, repeatability and linearity. Besides, it was robust to icterus, lipemia and hemolysis. The good sensitivity and specificity were obtained at 98% and 100%, respectively. NTAb results showed a high correlation with PRNT_50_ titers (*r* 0.61). Until July 2021, the BBIBP-CorV (76.3%) and Sinovac CoronaVac (20.5%) were the predominant vaccines injection in Shenzhen, China. Adolescent less than 18 years was the main unvaccinated group (52.1%). The seropositive rate of inactive SRAR-CoV-2 vaccines exceeded 97% after inoculation. The NTAb generated by Sinovac CoronaVac with the schedule of 0–56 days was found significantly lower than that by BBIBP-CorV (*P* < 0.001). The follow-up of NTAb changes in a cohort and the dynamic variation of NTAb in real world disclosed steep downward by almost three times for NTAb level occurred at three months post twice vaccinations. The seropositive ratio was at least 50% over 6 months.

**Conclusions:**

SARS-CoV-2 neutralizing antibodies assay show excellent analytical and clinical performances, and a high correlation with neutralizing activity. Anti-epidemic measures and the urgent trial of SARS-CoV-2 vaccine was calling for adolescents.

## Introduction

Globally, as of 20 January 2022, there have been 336,790,193 confirmed cases of COVID-19, including 5,560,718 deaths, reported to WHO. As things stand, future outbreaks of COVID-19 are likely to be sporadic and recurrent [1]. It is therefore imperative that the global population was acquired enough collective immunity by vaccines to avoid COVID-19 infection and disease progression. Meanwhile, a facile and reliable assay to assess the generated immune protection laid a foundation for a booster immunization due to increasing breakthrough infections.

SARS-CoV-2 virus is composed of a helical capsid formed by nucleocapsid (N) proteins bound to the single-stranded RNA, and an envelope made up of membrane (M), envelope (E) proteins and spike (S) protein. The S protein contains two functional subunits, S1 and S2. S1 consists of an N-terminal domain (NTD) and a receptor-binding domain (RBD) that is critical for determining tissue tropism and host range [2]. As of August 2022, a total of 85 commercially available serological kits had received an individual Emergency Use Authorization (EUA) by the U.S. Methods for detecting SARS-CoV-2 antibodies include fluorescent dual-functional lateral flow immunoassay [3], chemiluminescent (CLIAs) [4], enzyme-linked immunosorbent assays (ELISAs) [5] and other platforms. These kits generally detect IgM, IgG, or total antibodies to SARS-CoV-2 epitopes, including S1, S2, the RBD, or the N protein. The RBD protein is immunodominant and is the target of 90% of the neutralizing antibodies (NTAb) present in SARS-CoV-2 immune serum [6]. Therefore, the RBD have been widely selected as promising targets for vaccine development, as the detection target for immune effectiveness as well. In addition, vaccines against SARS-CoV-2 have been reported to produce NTAb comparable to those observed in naturally infected individuals [7, 8].

Generally, PRNT is accepted as the most specific test among conventional serological tests, this method remains the gold standard for serological testing and determination of immune protection. But the operation is complicated and the requirement of the experiment environment is very high. The rapid serological assays in daily detection with vaccination population and the interpretation are key pre-requisites of crucial importance in their efficacy. Therefore, the study aimed to report an analytical validation of rapid serological assays on CLIA, available on an automatic platform, and to describe the kinetics of NTAb in vaccinated participants.

## Materials and methods

### Subjects

**Fig. 1:**
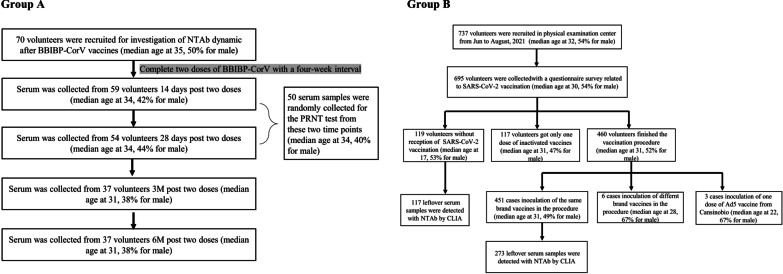
Characteristics of the whole population

Volunteers enrolled was composed of two Groups as Fig. 1. A longitudinal cohort study designed in the Group A aimed to evaluation of the analytical and clinical performances and the dynamic change of NTAb after inoculation of SARS-CoV-2 vaccines. The Group B was used for investigation of NTAb responses after vaccination in the real world. In Group A, 70 volunteers were recruited prior to BBIBP-CorV vaccines. Excluding the cases loss, 50 serum samples (median age at 34, 40% for male) were randomly collected for the PRNT test from 14-day and 28-day post two doses of vaccines. Then, the cohort of 37 vaccine recipients (median age at 31, 38% for male) were tracked with SARS-CoV-2 NTAb assay at prior to vaccination, one, three months and six months post two doses. In Group B, a total of 737 cases were recruited from physical examination center in Shenzhen Luohu People’ s Hospital (from Jun to August 2021) and 695 questionnaires related to SARS-CoV-2 vaccination was collected. The study protocol (2020-LHQRMYY-KYLL-033) was approved by the Ethics Committee of Shenzhen Luohu People’s Hospital. All the patients were informed and voluntarily agreed to participate, providing a written consent.

### Serological assays

In this study, a commercially available immunoassay was evaluated, the anti-SARS-CoV-2 NTAb detection kits (Shenzhen Mindray Biomedical Electronics Co., Ltd. Shenzhen, China). The above kit is based on a competitive chemiluminescence immunoassay (CLIA) that determines NTAb. All analyses were performed on Mindray CL-6000i automatic chemiluminescence immunoanalyzer, the cut off above 10AU/mL was identified as a positive criterion.

### Repeatability and intermediate precision evaluation

Precision estimation was performed with three serum pools at low, medium and high levels. The same pool aliquots were detected by quintuplicate measurements in five consecutive days. Nested analysis of variance (ANOVA) was used to estimate precision and the results were compared to those claimed by the manufacturer, following the CLSI EP15-A3 protocol [9].

### Linearity assessment

Quantitative determination of NTAb contributes to reveal the variation trend of immune response, hence the detection linearity and the range is necessary in serological assays. Linearity was assessed using serial dilution of two pools at high and low levels, as specified in the CLSI EP06-A: 2003 guideline (paragraph 4.3.1) [10]. Seven serial concentrations of NTAb were prepared by mixing a high-level at 417.29AU/mL and a low level at 0.60AU/mL with gradually diluted ratios. All measurements were performed in triplicate.

### Anti-interference ability verification

Interference testing was evaluated by the serum spiked with high concentrations of related interferent, as specified in the CLSI EP07-A2 [11]. Generally, the serological assay in automatic CLIA platform was interfered by hemoglobin, conjugated bilirubin and triglyceride-rich lipid. The artificial serum contained 500 mg/dL hemoglobin, 20 mg/dL conjugated bilirubin and 300 mg/dL triglyceride-rich lipid, respectively. The results were compared with and without the added interferents. All measurements were performed in triplicate.

### Plaque reduction neutralization test (PRNT)

PRNT test was performed with 50 samples after vaccination. Briefly, samples were heat-inactivated, incubated for 30 min at 56 °C, then diluted with DMEM medium containing 2% FBS by a factor of 10, 20, and 40, etc. The dilutions mixed with SARS-CoV-2 (isolated form a COVID-19 patient, 200 TCID50/ml) at a ratio of 1:1, were incubated at 37 °C for 1 h. The virus-serum mixtures were added into 24-wells plates (6 × 10^4^ Vero-E6 cells/well) and incubated at 37 °C for 1 h, in a 5% CO_2_ incubator. Then, the supernatant was removed and medium containing 0.8% sodium carboxymethylcellulose was added to each well. After 72 h of incubation, cells were fixed with a 10% paraformaldehyde (PFA) solution and stained with 0.5% crystal violet. After washing off the staining solution, the FFUs were counted after acquisition of pictures. The serum neutralization titer was defined as the reciprocal of the highest dilution resulting in a reduction of the control plaque count > 50% (PRNT_50_). The neutralization was calculated by Karber method. The formula is as follows. TCID50 is calculated in logarithms, and L is the logarithm of the lowest dilution of the virus. d is the group distance, that is, the dilution coefficient, S is the sum of the ratio of CPE to the number of inoculation in each group. The titer of 1:20 was identified as the seropositive threshold.

$$LogTCID50 = L + d\left( {S - 0.5} \right)$$

### Statistical analysis

The Graphpad Prism version 9.0 was applied to evaluate plaque reduction neutralization test results. ANOVA was used for precision, and polynomials was used for linear evaluation. The IBM SPSS Statistics v24 was employed to evaluate the assays’ clinical performances. Spearman’s coefficient was calculated to evaluate the correlation between variables. The Mann–Whitney U test and Kruskal–Wallis test were applied to analyze significance values of independent samples between two groups and multiple groups, respectively. *P*-values < 0.05 were considered to be statistically significant and the following denotations were used: *P ≤ 0.05; ns (not significant), *P* > 0.05.

## Results

### Repeatability and intermediate precision evaluation

Results for repeatability and intermediate precision of CLIA assay is reported in Table 1 according to the procedure suggested in CLSI EP-15-A3. Obtained data show acceptable intermediate precision of Mindray NTAb at low, medium and high levels, with CV values lower than those reported by the manufacturer.

**Table 1:** Precision results of SARS-CoV-2 NTAb obtained using a 5 × 5 design (quintuplicate measurement for 5 consecutive days) expressed as coefficient of variation (CV, %)

Measurand	Level, AU/mL	Design	Measured repeatability—CV, %		Intermediate precision—CV, %	
			Laboratory evaluation	Manufacturer’s claims	Laboratory evaluation	Manufacturer’s claims
NTAb	10.09	5 × 5	2.58	≤ 6.00	3.62	≤ 10.00
	24.62		1.26	≤ 6.00	1.95	≤ 10.00
	50.47		0.81	≤ 6.00	1.58	≤ 10.00

### Linearity assessment of SARS-CoV-2 antibodies

**Fig. 2:**
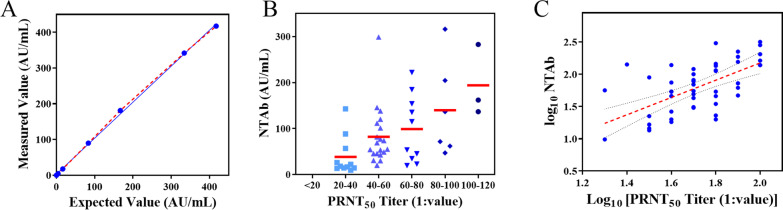
Performance analysis of SARS-CoV-2 NTAb. Linearity assessment of NTAb assays (**A**). Correlation between the CLIA results and PRNT_50_ titers serum antibodies in vaccine recipients (**B**, **C**)

Linearity for Mindray NTAb is investigated in Fig. 2A. The tested concentrations covered the manufacturers’ cut-offs. The measured results (Y) were observed a good linear relationship (Y = 1.007*X + 3.193, R2 0.999) with the expected ones (X) in the range of 2–400 AU/mL for SARS-CoV-2 NTAb.

### Anti-interference ability of SARS-CoV-2 NTAb on a CLIA platform

The concentrations of easily interfered substances including hemoglobin, conjugated bilirubin, and triglyceride-rich lipid added had exceeded the upper limit of medical reference range. As shown in Table 2, in comparison to serum without containing high interfered substances, the antibodies results in containing serum were found at ± 10% interfering bias, indicating serological assays on the CLIA platform had a high capability of anti-interference.

**Table 2:** NTAb analytical specificity studies with interference

Interferent	Low concentration sample			High concentration sample		
	Neat AU/mL	Spiked AU/mL	Interfering bias, %	Neat AU/mL	Spiked AU/mL	Interfering bias, %
Hemoglobin, 500 mg/dL	24.50	25.27	3.14	52.70	53.97	2.41
Conjugated bilirubin, 20 mg/dL	24.19	24.07	− 0.50	52.91	52.76	− 0.27
Triglyceride-rich lipid, 300 mg/dL	24.26	24.02	− 1.00	52.85	52.55	− 0.57

### CLIA result correlation with PRNT_50_ result

Fifty seropositive samples collected from vaccine recipients at 14 and 28 days post dose two were confirmed by PRNT_50_. Also, a total of sixty-six unvaccinated samples were selected for clinical performance evaluation. The relationship between NTAb on the CLIA platform and the PRNT_50_ results was shown in Fig. 2B, C. The concentration of NTAb by CLIA method scattered was closely related to the PRNT_50_ result. A positive correlation (Spearman’s r 0.61, P < 0.05) was found between the concentrations by CLIA and PRNT_50_ titers for serum antibodies in vaccine recipients. The diagnosis feature evaluated NTAb assay on the CLIA platform had a good sensitivity at 98% (95% CI, 87.99–99.89) and specificity at 100% (95% CI, 93.15–100.0), obtained a good positive predictive value (95% CI, 90.94–100.0) and negative predictive value (95% CI, 90.86–99.92).

### SARS-CoV-2 NTAb profile after vaccination in real world

**Fig. 3:**
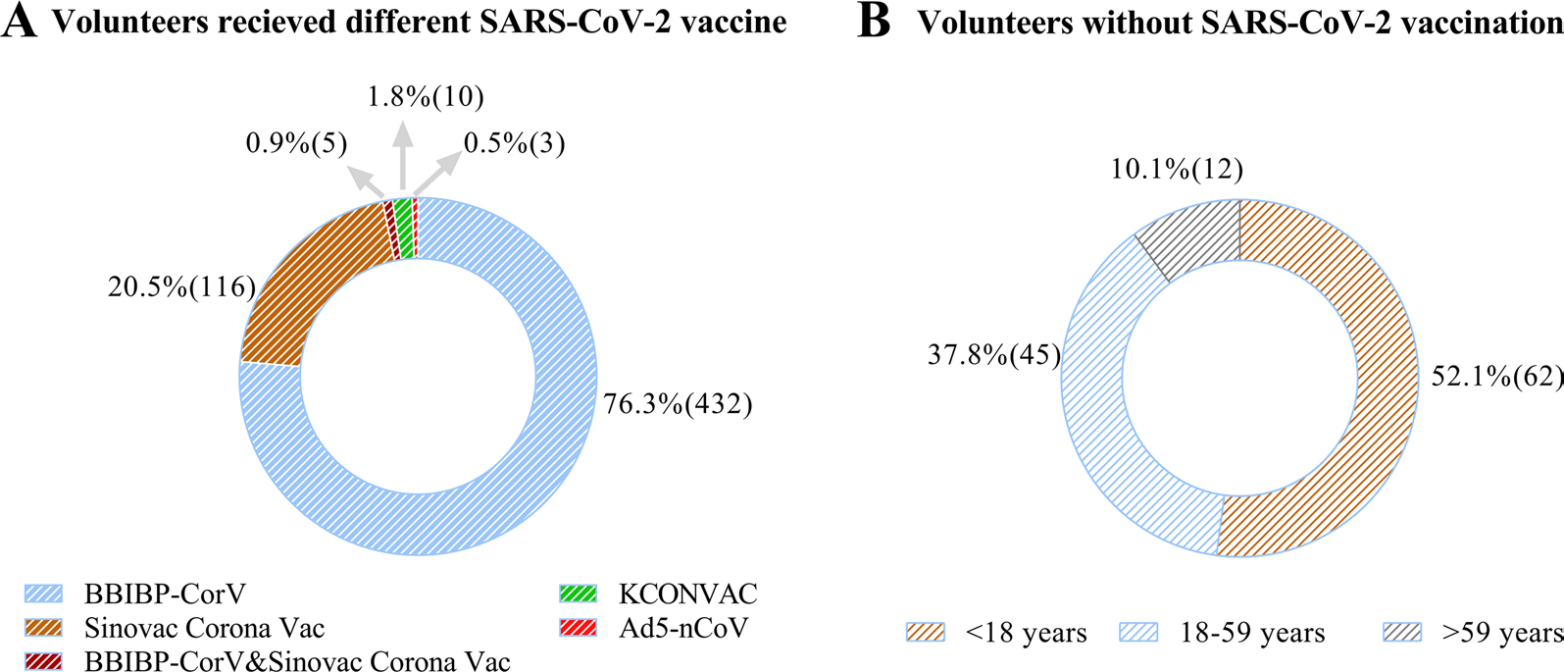
Analysis of vaccination in the real world. The percentage of volunteers received different SARS-CoV-2 vaccines in the real world, in Shenzhen, China (**A**). The proportion of subjects without inoculation of SARS-CoV-2 vaccines at three age groups (**B**)

In Shenzhen, China, a survey was performed on inoculation of SARS-CoV-2 vaccines and the SARS-CoV-2 NTAb detected by CLIA method in the real world. During the monitoring period, the percentage of 66% completed the vaccination covering different vaccine technology required with a single injection or two injections. There were 17% of subjects receipted only one injection and 17% did not get vaccinated against SARS-CoV-2. The ranking order of the number of injected vaccines from the highest to the lowest were BBIBP-CorV, Sinovac CoronaVac, cross inoculation with BBIBP-CorV and Sinovac CoronaVac, KCONVAC, Ad5-nCoV, shown in Fig. 3A. Especially, the proportion of BBIBP-CorV vaccines injection has exceeded half of the total number of volunteers. BBIBP-CorV vaccine was firstly officially authorized by CFDA in China and one of the largest production capacity of SARS-CoV-2 vaccines enterprises including Beijing, Wuhan, Chengdu and Lanzhou centers. Among the 119 subjects without vaccines injection, the main population was adolescent less than 18 years, accounting for 52.1% of the total (Fig. 3B). To build the colony immune defense, the adolescent aged from 3 to 17 years were injected with BBIBP-CorV or Sinovac CoronaVac in many districts of China Since August 2021.

Table 3 shows the differences in seropositivity and SARS-COV-2 NTAb levels between BBIBP-CORV and CoronaVac coronavirus vaccines, and whether there are differences between different immunization schedules. Four groups were compared by a Kruskal–Wallis test, the amount of NTAb produced by the vaccines and “prime-boost” strategies existed difference on the whole. AMann-Whitney U test was further performed between two immunization schedule and two vaccines, there was no remarkable difference among other groups except for BBIBP-CorV and Sinovac CoronaVac (0–56 days) (P < 0.001).

**Table 3:** Comparison of SARS-CoV-2 NTAb produced with different SARS-CoV-2 vaccines and immunization schedules

SARS-CoV-2 vaccine (an interval of two doses)	1 M		2 M		3 M	
	Median	SR	Median	SR	Median	SR
BBIBP-CorV (4 weeks)	70.49 (*n* = 10)	100%	78.43 (*n* = 11)	91%	29.17 (*n* = 15)	80%
BBIBP-CorV (8 weeks)	73.97 (*n* = 6)	100%	38.21 (*n* = 30)	87%	29.85 (*n* = 13)	77%
CoronaVac (4 weeks)	109.31 (*n* = 14)	100%	79.4 (*n* = 23)	96%	/	/
CoronaVac (8 weeks)	299.7 (*n* = 14)	100%	82.92 (*n* = 20)	100%	/	/
X^2^	8.977	/	12.344	/	/	/
*P*	0.03*	/	0.006*	/	0.235	/

SR represents seropositive rate; X^2^ represents the test statistic for Kruskal–Wallis test; *P* < 0.05 represents there was significant difference

**Fig. 4:**
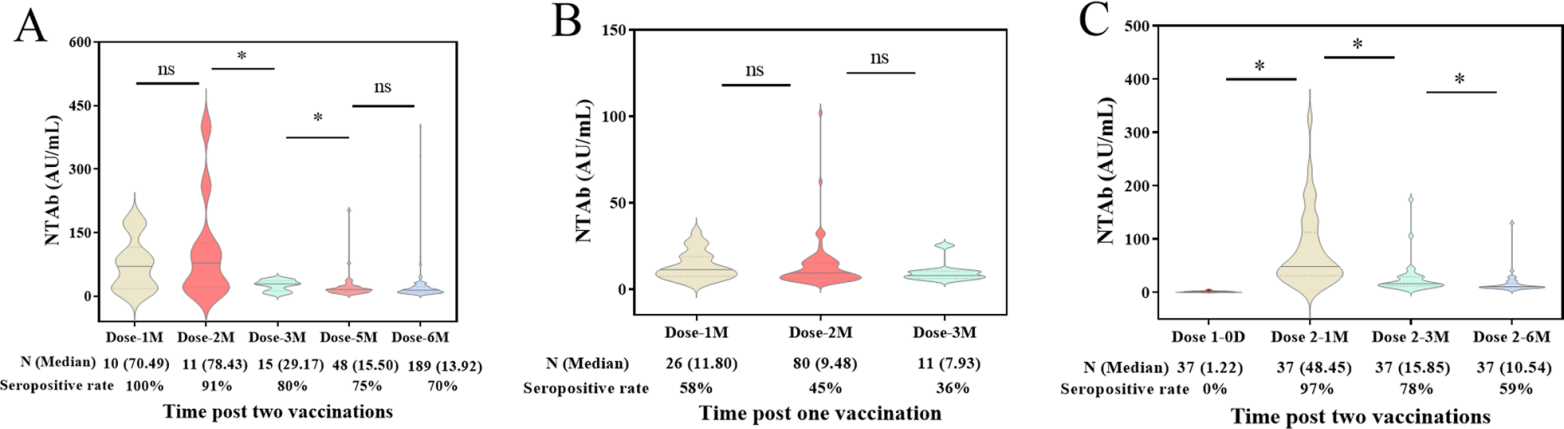
SARS-CoV-2 NTAb profile kinetics including antibody level and seropositive rate in the volunteers in Shenzhen, China. The group inoculation of two doses of BBIBP-CorV vaccines with a four-weeks interval (**A**). The group only inoculation of one dose of BBIBP-CorV vaccine (**B**). The group inoculation of two doses of BBIBP-CorV vaccines with a strict four-weeks interval and the volunteers engaged the dynamic SARS-CoV-2 NTAb monitoring from before inoculation to a half of year post two-vaccinations (n = 37) (**C**)

The seropositive proportion was gradually decreasing with the extending of vaccination time. As shown in Fig. 4A, the seropositive rate was 100% at 1 M after injection with two-doses of BBIBP-CorV vaccines (interval at four weeks) and the rate begun to fall two months later, but the NTAb level was not distinctly declined in comparison with a month ago. Until six months later, the seropositive rate remained 70%, and the level was decreased by five times compared to five months ago, dropping from more than 70 AU/mL to less than 20 AU/mL. For the population received only one dose of BBIBP-CorV vaccine, the seropositive rate was just 58% after one month later, and was less than twice the rate of two-doses procedure post three months (Fig. 4B). The results emphasized the necessity of the boosting dose of vaccine against SARS-CorV-2. A cohort consisting of 37 subjects was followed up for half of a year after inoculation of a two-doses of BBIBP-CorV vaccine with a 4-week interval to tracking the SARS-CoV-2 NTAb. The decreasing of seropositive rate and antibody level also were observed in the follow-up cases (Fig. 4C). Especially, a sharp drop was found in NTAb level from 1 to 3 M, afterward, the decline was significantly reduced.

## Discussion

Over the past year, numerous populations have received COVID-19 vaccine. How long the immune response generated and persisted against the SARS-COV-2 infection, have been always a concern. The serological assays for SARS-CoV-2 neutralizing antibody developed were the rapid approach for evaluation actual protective immunity of antibodies and laid a foundation for booster vaccination or interpretation the breakthrough infection [12].

Here we assessed analytical and clinical performances of a CLIA for Mindray SARS-CoV-2 NTAb, the correlation with PRNT_50_ results. Before the clinical performance validation and antibody variation profile studies, the method validation including precision, linearity and anti-interference ability verification were assessed following the CLSI EP15-A3, CLSI EP06-A and CLSI EP07-A3. The results show that Mindray CL-2000i has excellent precision characteristics. In fact, repeatability was < 3%, and intermediate imprecision was < 4%. The results allow us to reproduce the manufacturer's claims of precision at any concentration level. Linearity was performed in a range of values covering manufacturers’ cutoffs, the results obtained showed that is linear for NTAb immunoglobulins. We noted that the interferers triglyceride-rich lipid, conjugated bilirubin, and hemoglobin beyond the normal serum range (300, 20, and 500 mg/dL, respectively) had no effect on the quantitative detection of NTAb at low or high values. The test results (− 1.00% ~ 3.15%) were within the range of interfering bias declared by the manufacturer (≤ ± 10%). The CLIA assay has been extensively evaluated by the manufacturer, showing the clinical specificity of NTAb is 99.69% (95%CI: 99.38–99.84%) on 2549 specimens and the clinical sensitivity is 100% (95%CI: 91.24–100%) on 40 specimens that were demonstrated to be positive (≥ 1:20) using PRNT. The results showed that the sensitivity and specificity of CLIA for detecting NTAb was excellent.

It previously reported that antibody levels in COVID-19 convalescent were 2.65 times higher than in vaccinated individuals [13]. The variation of NTAb was also evaluated during a time interval previously recommended [14]. Tracking the variation of antibody profile after inoculation with COVID-19 vaccine, antibody levels decreased significantly at two months after vaccination. This is similar to the results of Julien Favresse et al. [15] reported that the immune response of participants vaccinated with BNT162b2 was maximal at 28 days and 42 days after vaccination, and significantly decreased at 56 days after vaccination. And the seropositive rate didn’t decrease during the monitoring period (90 days). But Bruna Lo Sasso et al. [16], the study found that the S-RBD IgG titer decreases significantly after the second dose of vaccine administration. This may have to do with different types of vaccines and individual's immune response [17]. In addition, our results showed that the antibody level and seropositive rate of participants after one vaccination were significantly lower than those after two vaccinations. Therefore, research into the durability of the long-period protection of various vaccines and the determination of the effect of enhanced dose to extend the duration of immunity against SARS-COV-2 infection is crucial. After six months of COVID-19 vaccination, there is an increase in the titer of neutrals and antibodies in humans [18]. Moreover, the increase of antibody titer in patients infected with novel coronavirus is more obvious and the protective effect against novel coronavirus is 20 to 47% [19, 20]. In addition, it has been reported that antibody remained stable up to 6 months after the onset of symptoms of COVID-19 patients, and then followed by a significant decline at 6–8 months [21]. But it is unclear how vaccine-induced antibody levels quantitatively compare to the natural SARS-CoV-2 infection. And whether vaccine-induced humoral immune persistence resembles natural infection has not been carefully defined.

The largest vaccination of BBIBP-CorV or Sinovac CoronaVac belonged to killed and complete virus-based vaccines. In reality, subjects were injected with the combination of “prime-boost” strategies such as 0–14 days (28/363, for BBIBP-CorV), 0–28 days (278/363) and 0–56 days (57/363). Our results showed there was no remarkable difference among other groups except for BBIBP-CorV and Sinovac CoronaVac (0–56 days). Much more subjects and the strict design of schedule was required for comparison the humoral immunity by different vaccines for preventing infection.

In our study, we observed that the CLIA NTAb results had a correlation (*r* 0.61) compared to PRNT_50_ titers. Andrea Padoan et al. reported a 0.689 correlation between RBD IgG and PRNT_50_ titers by using the Snibe diagnostics [22]. Andrea Padoan et al. reported VITROS anti-S1 total antibody (R^2^adj = 0.117) and Elecsys anti-N IgG (R^2^adj = 0.046) showed a very limited association with PRNT_50_ titers [23]. Livia Mazzini et al. [24] showed the excellent agreement between the ELISA VM_IgG_RBD and MNT, with coefficients of 0.83. Thus, antibodies targeting the RBD binding domain of S protein have the strongest correlation with gold standard results. This further confirms that the protective effect of antibodies to the RBD domain is extremely important [6, 25, 26]. A proportion of patients may have high titers of binding antibodies but negative NTAb also should be concerned, whether because the vaccine induced antibody responses are heterogeneous or intrinsic mechanism is existed and required further studies.

In conclusion, the data reported in this study showed that Mindray anti-SARS-CoV-2 NTAb assay achieve excellent analytical and clinical performances. Until July 2021, the BBIBP-CorV and Sinovac CoronaVac were the predominant vaccines injection in Shenzhen, China. Adolescent less than 18 years was the main unvaccinated group, the anti-epidemic measures and the urgent trial was calling for such population. The NTAb generated by Sinovac CoronaVac with the schedule of 0–56 days was found significantly lower than that by BBIBP-CorV. The follow-up of NTAb changes in a cohort and the dynamic variation of NTAb in real world disclosed steep downward for NTAb level occurred at three months post twice vaccinations. Fortunately, the seropositive ratio was at least 50% over 6 months, the cell-mediated immune responses combined with the humoral immunity defended the body against the infection of SARS-CoV-2. The limitation of the study is that the sample size is insufficient. The further study should be conducted by increasing sample size to explore the rule of NTAb response in the real world. The continuing follow-up with the vaccine cohort was required. The study summarized the antibody kinetics model and provided a basis for whether to vaccinate a third booster needle, and in cases of vaccine shortage prioritize their administration to seronegative people. Moreover, the protective effect of antibodies against the RBD domain of S protein was confirmed.

## Data Availability

The datasets generated and/or analyzed during the current study are not publicly available due to the institutions policy to code and archive data in a central repository of the hospital, but are available from the corresponding author on reasonable request.
